# A Review of the Role of Artificial Intelligence in Healthcare

**DOI:** 10.3390/jpm13060951

**Published:** 2023-06-05

**Authors:** Ahmed Al Kuwaiti, Khalid Nazer, Abdullah Al-Reedy, Shaher Al-Shehri, Afnan Al-Muhanna, Arun Vijay Subbarayalu, Dhoha Al Muhanna, Fahad A. Al-Muhanna

**Affiliations:** 1 Department of Dental Education, College of Dentistry, Deanship of Quality and Academic Accreditation, Imam Abdulrahman Bin Faisal University, Dammam 31441, Saudi Arabia; 2 Department of Information and Technology, Imam Abdulrahman Bin Faisal University, Dammam 31441, Saudi Arabia; 3 Health Information Department, King Fahad hospital of the University, Al-Khobar 31952, Saudi Arabia; 4 Department of Information and Technology, Family and Community Medicine Department, Family and Community Medicine Centre, Imam Abdulrahman Bin Faisal University, Dammam 31441, Saudi Arabia; 5 Faculty of Medicine, Family and Community Medicine Department, Family and Community Medicine Centre, Imam Abdulrahman Bin Faisal University, Dammam 31441, Saudi Arabia; 6 Breast Imaging Division, Department of Radiology, Imam Abdulrahman Bin Faisal University, Dammam 31441, Saudi Arabia; 7 Radiology Department, King Fahad hospital of the University, Al-Khobar 31952, Saudi Arabia; 8 Quality Studies and Research Unit, Vice Deanship of Quality, Deanship of Quality and Academic Accreditation, Imam Abdulrahman Bin Faisal University, Dammam 31441, Saudi Arabia; 9 NDirectorate of Quality and Patient Safety, Family and Community Medicine Center, Imam Abdulrahman Bin Faisal University, Dammam 31441, Saudi Arabia; 10 Nephrology Division, Department of Internal Medicine, Faculty of Medicine, Imam Abdulrahman Bin Faisal University, Dammam 31441, Saudi Arabia; 11 Medicine Department, King Fahad hospital of the University, Al-Khobar 31952, Saudi Arabia

**Keywords:** artificial intelligence, ethics, governance, healthcare

## Abstract

Artificial intelligence (AI) applications have transformed healthcare. This study is based on a general literature review uncovering the role of AI in healthcare and focuses on the following key aspects: (i) medical imaging and diagnostics, (ii) virtual patient care, (iii) medical research and drug discovery, (iv) patient engagement and compliance, (v) rehabilitation, and (vi) other administrative applications. The impact of AI is observed in detecting clinical conditions in medical imaging and diagnostic services, controlling the outbreak of coronavirus disease 2019 (COVID-19) with early diagnosis, providing virtual patient care using AI-powered tools, managing electronic health records, augmenting patient engagement and compliance with the treatment plan, reducing the administrative workload of healthcare professionals (HCPs), discovering new drugs and vaccines, spotting medical prescription errors, extensive data storage and analysis, and technology-assisted rehabilitation. Nevertheless, this science pitch meets several technical, ethical, and social challenges, including privacy, safety, the right to decide and try, costs, information and consent, access, and efficacy, while integrating AI into healthcare. The governance of AI applications is crucial for patient safety and accountability and for raising HCPs’ belief in enhancing acceptance and boosting significant health consequences. Effective governance is a prerequisite to precisely address regulatory, ethical, and trust issues while advancing the acceptance and implementation of AI. Since COVID-19 hit the global health system, the concept of AI has created a revolution in healthcare, and such an uprising could be another step forward to meet future healthcare needs.

## 1. Introduction

Health systems worldwide are at a crossroads and face exponential healthcare cost developments that have far outpaced GDP growth rates to support health system sustainability [1]. This matter was very straightforward with the emergence of the 2019 coronavirus disease (COVID-19) pandemic and the Ukraine war. There is a combination of tight finances, elderly inhabitants, rising chronic diseases, and the strain on healthcare systems that previously struggled to cope with increased demand for service accessibility and availability. In addition, the COVID-19 pandemic is leading to health system failure in some countries, e.g., India, Brazil, and Indonesia [2].

As health systems depend on firm disease management pathways and evidence-based care tactics to meet needs and regulate practices according to industrial healthcare delivery services, the concept of “HRO” emphasizes a Highly Reliable Organization (HRO) by having its services managed by either an “accountable care organization (ACO)” or a “health maintenance organization (HMO)” [3]. However, the incidence of chronic diseases in the United States (USA) is steadily increasing, i.e., 60% of adults have one chronic disease, and 40% have more than two chronic diseases, totaling USD 3.3 trillion in annual healthcare costs [4]. In addition, this picture quickly changed with the emerging infectious disease, which was first reported in Wuhan, China, in 2019 and designated as COVID-19 by the World Health Organization on 11 February 2020 [5]. Since then, healthcare has been undergoing a digital transformation that will transform many of the fundamental building blocks of medical care [6]. This condition might be due to the massive pressure exerted by COVID-19 on global healthcare and the associated infrastructure, supply chain, and employees. The pandemic also forced healthcare stakeholders to adopt digital technologies [7,8]. Notably, key foundational changes occurred in the healthcare sector during the post-pandemic. For instance, current-generation customers (patients) showed active involvement in healthcare-associated decision making due to the increased acceptance of virtual healthcare systems and associated digital innovations [9]. However, protuberant challenges can occur, and the strategies to overcome them would create a way for the voyage to reach the upcoming healthcare era. Patients and their experiences and needs motivate innovations in the healthcare sector. Their main inclinations comprise the creation of digitally empowered physician–patient interactions, confirming the provision of patient-centric amenities across the globe [10]. The necessity for deploying advanced digital devices has become a requirement to offer augmented customer satisfaction, permitting tracking, checking the health status, and better drug adherence to be achieved [11]. Such aspects would be more beneficial during the post-hospitalization period using digital health platforms. At the same time, healthcare customers are cautious about sharing their confidential data; hence, healthcare organizations (HCOs) are in place to preserve the customer’s trust by representing transparency, empathy, and reliability in their services [11]. 

The rise of biomedical science, including genomics, digital medicine, artificial intelligence (AI), and its subset, namely, machine learning (ML), provides the backdrop to healthcare transformation, with novel emerging technologies, and there is a prerequisite of a new type of labor force and standard of practice. Genomics and other technologies, including biometrics, tissue engineering, and the vaccine industry, can improve and transform diagnostics, therapeutics, care delivery, regenerative treatment, and precision medicine models [12]. Table 1 describes the definitions of terms related to AI.

Moreover, digital health technologies (DHTs) comprise mobile health (mHealth), health information technology (HIT), wearable devices, telehealth, telemedicine, mobile Internet devices (MIDs), and personalized medicine [37], and recently, innovative technological advancements that are influencing smart health are AI, metaverse, and data sciences [38]. These technologies lead to better prevention; the early detection of fatal diseases; and the remote management of chronic diseases peripherally to the customary care locations, such as wirelessly observed therapy (WOT), following a novel method of monitoring adherence to therapy [39]. The most promising new way is to offer and deliver health services anywhere and at anytime in the age of disruptive and minimally invasive medicine. MIDs allow the recipient to access important resources, including associated applications and social media (SM). The available applications of MIDs are numerous and provide access to scientific databases such as Medscape, Web of Science, and Scopus to professionals. While SM networks, namely, YouTube, Facebook, WhatsApp, Wikipedia, and other instant messaging applications (IMAs), can be available for both professional and non-professionals. Such digital health modalities using AI in healthcare are accelerating in the post-COVID-19 era [40]. 

AI, ML, and DHT have fueled a revolution in healthcare, especially since COVID-19 crippled the global healthcare system. In particular, AI is presently integrating new technologies, such as the Internet of Things (IoT), into the DHTs used by consumers. As AI and ML are widely implemented in healthcare systems, the IoT is expected to transform into the intelligence of things [1]; how the collected data are utilized to alter processes would swing behavior and values [41]. In addition, intelligent medical technology, i.e., powered AI, has been met with some enthusiasm among ordinary people, because it makes the 4P model of medicine, namely, predictive, preventive, personalized, and participatory, and thus patient autonomy, possible [42]. Integrating AI into healthcare has already been shown to lead to better, faster, and lower-cost healthcare [12].

**Figure 1:**
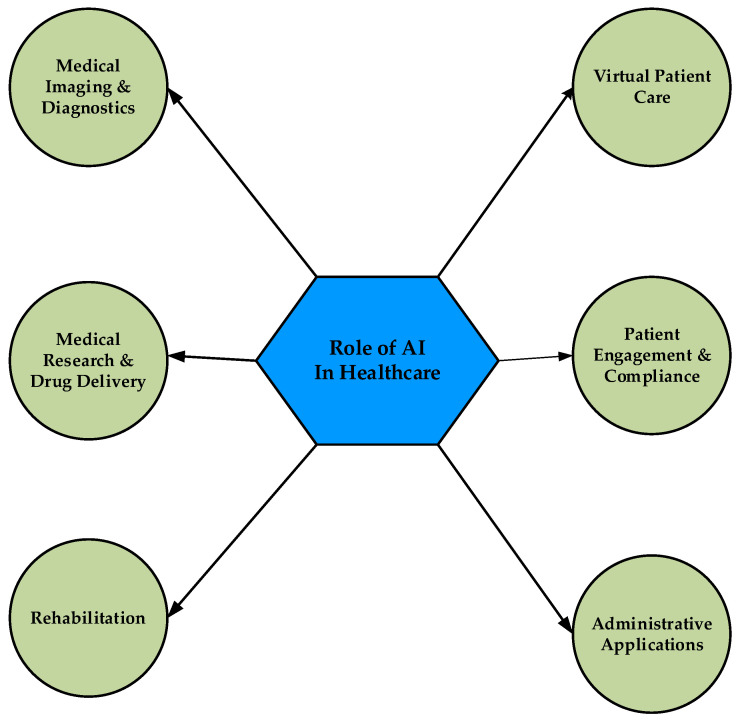
Application of AI in various aspects of healthcare.

Digital health tools offer healthcare providers a more holistic view of patient health by allowing providers to access patient data. They also allow patients to be provided with more statistics about their health by their physicians. These offer real opportunities to improve therapeutic outcomes and efficacy, although there are concerns that such modalities may have greater psychological impact, particularly with the widespread use of SM and IMAs by patients, the public, and professionals [43]. Additionally, accumulated data from multiple sources, such as health information systems (HISs), wearable devices, telemedicine, mHealth, telehealth, MIDs, and other AI-powered medical technology [44], create big data that accelerate the utilization of ML and AI in health systems using the learning process from the data these sources obtain, including data from research information, user experience, and the investigation of big datasets [45]. Further, electronic health records (EHRs) encompass various healthcare data of patients. Such health datasets can be connected using novel AI technologies to gain accurate insights into patient care. AI has also emerged as a choice for big data applications in healthcare [46]. Moreover, big data analytics enables healthcare providers to improve their clinical services by improving EHRs using analytical algorithms [47]. Such analytics also uses the advancement of AI to filter big data on various grounds for better data analysis [48]. As AI is widely used in various areas of healthcare to improve patient health outcomes and provide healthcare at lower cost, this review aims to reveal its role in healthcare, focusing on the following key aspects (Figure 1): (i) medical imaging and diagnostics, (ii) virtual patient care, (iii) medical research and drug discovery, (iv) patient engagement and compliance, (v) rehabilitation, and (vi) other administrative applications. In addition, the authors address some challenges to using AI in healthcare. These results complement the existing literature to take the benefits of AI tools one step further in healthcare. 

## 2. Role of AI in Healthcare 

### 2.1. Medical Imaging and Diagnostic Services

AI is a powerful tool for image analysis that is increasingly being used by radiology professionals for the early diagnosis of different diseases and for reducing diagnostic errors in the context of prevention. Likewise, AI is a smart and potential tool for analyzing ECG and echocardiography charts that cardiologists use to support their decision making. The Ultromics platform, which was reported in a hospital in Oxford, utilizes AI to analyze echocardiography scans that sense heartbeat patterns and detect ischemic heart disease [49]. AI has presented encouraging results in the early detection of diseases such as breast and skin cancer, eye disease, and pneumonia using body imaging modalities [50,51,52]. AI tools analyze speech patterns to forecast psychotic occurrences, and recognize and screen the features of neurological diseases such as Parkinson’s disease [53,54]. A recent study predicted the onset of diabetes using ML models. The results showed that a two-class augmented decision tree was the best model to predict the different variables of diabetes [55]. Furthermore, Gudigar et al. [56] stated that several medical imaging tools, including X-ray, computed tomography (CT), and ultrasound (US), applying AI techniques have significantly contributed to combating COVID-19 by aiding in early diagnosis. Their results reported that all handcrafted feature learning (HCFL), deep neural networks (DNN), and hybrid methods were able to predict COVID-19 cases. A recent review also explained in detail the use of CT scan, X-rays, MRI, and ultrasound in diagnosing COVID-19. It stated that AI has been instrumental in helping the public fight against the dreaded virus [57]. Moreover, a deep learning model named transformer is used in medical imaging analysis and includes registration, detection, classification, image-to-image translation, segmentation, and video-based applications [34]. Previous studies explained the application of transformers in differentiating COVID-19 from pneumonia using X-ray and CT images to meet the severe prerequisite to quickly and efficiently manage COVID-19 cases [58,59]. Another study applied the ImageNet-pretrained vision transformer (ViT)-B/32 network to detect COVID-19 using inputs such as patches of chest X-ray images [60]. A study by Wang et al. [61] proposed a new hybrid chest CT-built method to automatically detect COVID-19. It is a computer vision-based diagnosis technique based on wavelet Renyi entropy (WRE) and a proposed three-segment biogeography-grounded optimization (3SBBO) algorithm. It comprises WRE, a feedforward neural network (FNN), and the 3SBBO algorithm. WRE extracts image features; 3SBBO optimizes the biases and weights of the network; and the FNN classifies the images. This method showed better performance than kernel-based extreme learning machine, extreme learning machine with bat algorithm, and radial basis function neural network in detecting COVID-19. Additionally, Gheflati et al. [62] reported that the ViT is used to sort normal, malignant, and benign breast tissues based on ultrasound (US) images. It showed better efficacy in the classification of US breast images than convolutional neural networks (CNNs).

Furthermore, AI comprises the application of artificial neural networks, i.e., deep learning techniques named Generative Adversarial Networks (GANs), that impact the field of radiology. GANs contain two artificial neural networks, i.e., (i) a generator that synthesizes images similar to real images, and (ii) a discriminator that reveals the difference between synthetic and real images. Concerning radiology, the generative model can duplicate the images consistent with training and synthesize new images with the features of the images in the training dataset. The discriminant model is trained to classify the images, for example, whether a radiograph displays or not pneumonia. It was concluded that the generator model trained together with the discriminator model can result in progresses in radiological activities such as abnormal detection, image synthesis, and cross-domain image synthesis [63]. Skilled radiologists found it hard to distinguish between lung cancer nodules images generated by GANs and real images [64]. In addition, GANs offer an excellent opportunity to improve medical education and research. They swiftly develop training material and simulations for student learning. For instance, when students find it hard to differentiate “lower lobe collapse” and “consolidation,” samples of each type could be developed and displayed to them. Thus, synthetic data can support student learning by presenting edge-case learning resources. Furthermore, synthetic control arms have been developed by modeling placebo groups grounded on historical information, and those arms reduce the requirement of a real-life placebo group, thereby reducing costs and expanding the count of treatment arms in clinical trials [65]. Additionally, ChatGPT is a deep learning-based large language model used by the public for medical advice; thereby, it has turned into a basis for concern. Substituting professional medical advice, the public could be tempted to use such a model to determine possible diagnoses based on clinical features or gain treatment suggestions [66]. A previous US-based study found that about one-third of adults required Internet-based medical advice for self-diagnoses. Subsequently, about half of them consulted a doctor about the Internet-based outcomes [67].

Apart from this, AI-based medical practice, mainly medical imaging-guided diagnosis and therapy, is facilitated by a metaverse of ”medical technology and AI” (MeTAI). The critical applications of MeTAI include “virtual comparative scanning”, “raw data sharing”, “augmented regulatory science”, and “metaversed medical intervention”. A model execution of the MeTAI ecosystem is described as follows: The patient’s scans are first simulated using virtual machines to reveal the best imaging outcome before he/she undertakes an actual CT scan. Based on this knowledge, a real scan is made. Subsequently, the metaverse images are shared with the patient’s physicians’ squad after obtaining the patient’s approval. Following security procedures, the tomographic raw data and images are shared with the medical researchers. The collection of real and simulated images, data, and other medical evidence can be combined in the metaverse and applied in augmented clinical trials. Lastly, the patient is exposed to a metaverse-assisted remote robotic operation and followed up in the metaverse for rehabilitation if it is therapeutically advised. However, MeTAI experiences challenges such as security, disparity, investment, and privacy [68]. 

Additionally, medical scans are systematically gathered and saved for some time and are freely obtainable to train AI systems [69]. Those AI systems could reduce the time and cost of examining medical scans and potentially allow more scans to be taken for superior targeted management [70]. AI is also impacting clinical decision making and disease diagnosis. It can process, analyze, and report a large number of data across different modalities for disease diagnosis and clinical decision making. It can support physicians to make better clinical decisions or even replace human decisions in therapeutic zones [71]. Furthermore, investigations leveraging computer-aided diagnostics have presented outstanding sensitivity, accuracy, and specificity in uncovering minor radiographic deviations, with the capacity to advance public health. Nonetheless, outcome assessment in AI imaging studies is usually described as lesion detection, ignoring the biological severity and nature/type of a lesion, which could give a skewed picture of AI output. In addition, applying non-patient-related radiological and pathological endpoints could increase the expected sensitivity at the cost of increasing false positives and possibly overdiagnosing by detecting minor abnormalities that might mimic subclinical disease [72].

### 2.2. Virtual Patient Care

Baig et al. [73] noted that the advancement of wearable technology and the potential of using ML and AI in healthcare is an idea that has already been explored. Thus, patient monitoring and management via virtual care with active and sensible wearable technology solutions have become a reality and part of the standards of care. In addition, AI plays a role in controlling chronic diseases such as diabetes mellitus, hypertension, sleep apnea, and chronic bronchial asthma using wearable, non-invasive sensors. [74]. A previous study recommended a smart sensor system based on a combined sensor network to observe a person’s home and environment and obtain data on a person’s health status and behavior. The recommended platform includes sensors that are unobtrusive, biomedical, and wearable. These sensors monitor physiological variables such as respiratory rate, pulse rate, breathing waveform, blood pressure, and ECG. A smart device (such as a tablet) has been proposed to act as an interface between the person and the sensors. In addition, the collected data are sent to the cloud for storage and data analysis for elderly care [75]. A case report by Patel and Tarakji [76] also discussed a patient in whom atrial fibrillation was positively detected as the probable cause of her stroke following a broad negative examination. The patient was warned to record ECG signals using a wearable digital device. Her electrophysiologist later confirmed those recorded signals. Thus, consumer wearable digital devices support reaching the precise diagnosis. Regarding mental health disorders, Sukei et al. [77] demonstrated the possibility of building ML models for predicting emotional states using mobile sensor data that can handle diverse data with large amounts of missing information. Such models could provide valuable tools for physicians to assess patients’ mood states. Further research is recommended to find a solution for sparse and lost tagged data so that future focus can be placed on developing more innovative models. 

Since the prevalence of SARS-CoV-2 has caused the COVID-19 pandemic worldwide, progress has been observed in wearable devices that measure physiological changes in biometrics or even transmit online active patient monitoring [78]. Bogu and Snyder [79] suggested that wearable sensor data could be used as indicators for the early prediction of COVID-19, and with real-time wearable research on COVID-19 cases, the clinical features glossed over by users and justified by laboratory investigations will continue to improve knowledge related to tracking and detecting COVID-19 outbreaks. AI using predictive modules with ML and big data can help predict the progression of some diseases, such as diabetic nephropathy, and even diagnose SARS-CoV-2 infection in solid organ transplantation [80]. Yu et al. [81] emphasized the importance of integrating AI into bedside care in COVID-19 and the coming pandemic after examining encounters in emerging AI-enabled applications for point-of-care use in such events. In addition, the necessity of remote healthcare services has been created due to the COVID-19 pandemic. Metaverse applications can deliver a better experience than traditional videoconferencing-grounded telemedicine applications [82]. A recent study stated that the growth of telemedicine with metaverse development increased 38-fold during COVID-19 [68]. Such growth could have been due to the decrease in face-to-face consultations and the control of the risk of spreading the virus during the COVID-19 pandemic [83,84]. It also revealed the possibility of new metaverse tools, namely, virtual comparative scanning and raw data sharing, which would be consistent, easy to use, and inexpensive, and would work well [68]. Additionally, metaverse systems could utilize augmented reality (AR) glasses so that the users can access live videotapes and audio chats to interact with clinicians in real time. AR solutions would allow users to connect directly and offer a live flow of emergency circumstances for remote physicians to deliver on-time, faster, and on-spot management [85]. The application of current technologies such as AI, telepresence, blockchain, virtual reality (VR), augmented reality (AR), and digital twinning provides the experience of innovative means for offering low-cost management that improves patient outcomes. The metaverse develops a virtual world experience using the Internet in which human emotions and motions are simulated. It comprises the comprehensive financial and social constructs of both real and virtual environments [86]. Furthermore, AI could aid in reinforcing the metaverse structure to improve the 3D immersive experience and enhance the essential services of virtual worlds [87].

Remote patient monitoring (RPM) is a subset of telehealth, and it permits HCPs to monitor, investigate, and report patient conditions away from the traditional location. RPM smooths the performance of medical action using sensors and communication technologies. It makes it easier to remotely examine health data or patient issues. It also allows patients to engage and recognize their health condition [88]. The reliability of conventional patient-monitoring structures hinges on HCPs’ time management, which relies on their workload. Such surveillance also includes invasive methods requiring skin contact to screen health status. RPM in healthcare is achieved by integrating novel IoT methods: contact-based sensors, wearable devices, and telehealth applications. It is often applied to examine vital signs or other physiological variables such as the recognition of motion, which can support medical judgment or therapeutic regimens for diseases such as psychological illnesses and movement disorders [89]. Additionally, healthcare providers leveraged RPM platforms to facilitate the continuity of patient care during the COVID pandemic. A recent study evaluated two remote patient-monitoring platforms, the CareSimple COVID platform and the Telecare COVID platform, for monitoring COVID-19 patients. These two platforms were reported to have been well received by COVID-19 patients, with minimal significant discrepancies in patients’ experiences of the two platforms. It is recommended to consider using these platforms in a post-pandemic and post-hospitalization period [90]. Concerning RPM applications, conventional ML and deep learning are commonly used AI technologies to sense and forecast vital signs and classify patients’ physical movements. AI-powered RPM designs have transformed healthcare monitoring applications to detect early patient deterioration, absorb patient behavior patterns using reinforcement learning, and personalize the monitoring of patient health variables using federated learning. However, AI can transform RPM facilities, but it has some challenges, such as privacy, signal processing, data volume, uncertainty, imbalanced datasets, feature extraction, and explainability [89].

Moreover, ChatGPT, an AI language model, was developed by OpenAI. It functions as a more accurate AI-powered chatbot that can understand natural language conversations and respond to user queries. The ChatGPT-powered chatbot delivers information about a particular medical disease or management regime. It delivers precise and current replies to the patient’s questions concerning their clinical features, prescription drugs, and therapeutic procedures in various languages. It outlines patients’ medical information for HCPs and may aid them in performing RPM to sustain patient health. Further, it reminds patients to check their vital signs so that they can alarm HCPs if any abnormal changes occur. It allows patients to fix their appointments with physicians [91]. ChatGPT might also offer responses for a computer program that helps patients handle their treatment, similar to a virtual assistant who alerts them to follow their medical prescription and provide information about their health status. The growth of virtual assistants for patients is an example of how ChatGPT is applied in medicine. A virtual assistant may counsel in treating a chronic disease such as diabetes or prescribe over-the-counter drugs or home medicines to flu or cold patients. Digital platforms such as mobile applications, voice assistants, and websites may be applied to access these virtual assistants. However, ChatGPT in healthcare has limitations, namely, issues related to medical ethics, data interpretation, privacy, security, consent, and liability [91].

On the other hand, data connectivity is a disadvantage of installed Wearable Patient-Monitoring (WPM) systems, where patients are secured inside fixed spaces with low-Bluetooth-range monitoring devices. In addition, end-user acceptance is a crucial aspect of WPM systems. It relies on user awareness, and patient and physician acceptance. Cost issues can arise when using mobile data for communication over time and different data collections [73]. 

### 2.3. Medical Research and Drug Discovery

AI is ideally suited to analyze the large and complex datasets used in medical research [92]. In addition, it can be applied to hunt scientific research works, integrates various types of data, and supports drug innovation [93]. Pharmaceutical agencies are focusing on AI to streamline drug development. Scientists can utilize predictive analytics to recognize appropriate aspirants for clinical trials and develop exact models of biological processes [92]. ML contributes to clinical trials in the pre-trial phase, choosing the cohort, organizing of participants, and collecting and analyzing data. It can augment the patient-oriented view, generalizability, efficacy, and achievement of clinical trials. However, ML needs more emphasis on its functional and philosophical obstacles in clinical trials. In addition to ML, natural language processing (NLP) has exposed potential across various actions enhancing participant management in clinical trials; however, these tools’ effects on clinical trial quality and participant experience are uncertain. Further research can be conducted comparing different methods to improve participant management [94]. In clinical research, generative AI may be used to create synthetic data to enhance datasets and increase diversity [65]. Additionally, researchers can perform trials in an immersive and controlled setting using metaverse applications. The benefit of applying the metaverse is aiding research collaboration among the researchers who are physically distant. The metaverse permits them to research together in a virtual space similar to researchers that are in the same chamber [95]. Another AI-based tool, ChatGPT, may be used in clinical trials to support data collection and provide information about clinical trials [91]. It can aid in condensing pertinent publications and recognizing significant results, supporting medical researchers to competently navigate massive Internet-based evidence [96]. In addition, a chatbot employing ChatGPT aids in translating the medical language for medical researchers. Nevertheless, more ethical issues may arise when using chatbots in medical research [91].

Moreover, AI technologies in drug discovery have developed from ML, bioinformatics, and cheminformatics models [97]. These technologies can dramatically decrease the high cost and time needed for new-drug discovery [98]. An earlier study stated that an AI-based robot scientist (Eve) performed the drug development process rapidly and economically [99]. Concerning drug discovery, AI is mainly used to search for candidate molecules, but it is probable that it could be dynamically used in drug discovery in the future [98]. Many drug discovery successes supported by AI indicate the capability of AI-built-in firms of rapidly exploring drug candidates. For instance, Toronto-based deep genomics utilized an AI workbench platform to create a new genetic target and the respective oligonucleotide drug candidate DG12P1 for managing an unusual, inherited form of Wilsons’ disease [97]. Furthermore, recognizing new drug targets is crucial while conducting drug discovery research for finding new first-in-class clinical drugs [97]. AI can detect hit and lead compounds and offer the rapid authentication of the drug target and the better scheming of drug structure design [100,101]. The capability of AI of forecasting the interaction between drug and target was also utilized to aid the repurposing of current drugs and to evade polypharmacology. Repurposing a current drug passes it toward subsequent phases of clinical trials [100]. Previous studies stated that ChatGPT might be applied to assess a large body of scientific evidence, comprising patents and research publications; thereby, new drug targets and creating innovative ideas are determined. In the case of drug development, it helps train the model on a massive volume of scientific evidence before the model is used to deliver assumptions or suggestions for further research [102,103]. Apart from this, AI is used for drug screening [104]. Previous studies stated that various algorithms, namely, extreme learning machines, DNNs, random forest (RF), support vector machines (SVMs), and nearest-neighbor classifiers, are applied for virtual screening (VS) grounded on synthesis viability and for forecasting in vivo toxicity and activity [105,106]. 

Notably, reviewing the proteins constituting a virus (spike protein) is the function of AI in vaccine development. The categorization of numerous components in a complicated structure can be feasible using an AI system to determine the one that most probably elicits a robust immunological response [107]. Further, the evolution of AI systems in healthcare supports the innovation of the genomic series of the COVID-19 virus and its variants. It also aids in developing vaccines and drugs (comprising drug repurposing) to obtain active preventive and healing agents for restraining the COVID-19 pandemic [108]. 

### 2.4. Patient Engagement and Compliance

Patient engagement and compliance is healthcare’s ‘last stretch’ issue and the end blockade between better and poor health outcomes. Non-compliance means when a patient fails to shadow a treatment course or take the recommended medications. If patients are highly engaged in healthcare, their health outcomes are probably better, i.e., healthcare utilization, cost, and patient experience [109]. A healthcare leaders and executives survey reported that less than 50% of their patients were highly involved in treatment plans [110]. Healthcare providers use their clinical experts to develop treatment plans to improve patients’ acute or chronic health. Nevertheless, mostly, it does not matter when a patient misses the required behavioral changes, such as controlling weight, scheduling a follow-up visit, and obeying a treatment plan [109]. Such conditions raised the implementation of AI to successfully enhance patient engagement. ML and workflow engines are progressively being applied to driving composite interventions and the care spectrum [111]. Messaging alarms and appropriate custom-made material that encourage behavior at moments is the auspicious pitch of research [109]. 

Moreover, a study reveals that leveraging apps and online portals facilitating patient communication with HCPs can improve engagement rates by up to 60% and above. Healthcare apps collect, save, and distribute patient data on the cloud. These apps also permit users to access data wherever and whenever they want and possess the capacity to enhance a patient’s health outcomes. These are AI-based apps for medical consultation that let patients obtain info (non-emergency). Certain apps have also been given the ability to follow up with the patient and give alarms for drug intake [112]. Furthermore, ChatGPT is being utilized in various healthcare apps, including in automating long tasks such as summary, note writing, and report production, thereby making those tasks time-saving and more efficient. It assists patients in checking symptoms, fixing appointments, and managing drugs, aiding patient compliance and education, and the self-management of chronic diseases [91].

### 2.5. Rehabilitation 

AI has innovative applications in the field of rehabilitation. It is an idea that includes physical (robotics) and virtual (informatics) branches. Additionally, a subset of AI known as ML refers to precise methods for building algorithms that naturally improve with practice. In rehabilitation, ML is used for perioperative medicine, brain–computer interface technology, myoelectric control, symbiotic neuroprosthetics, etc. ML methods have also been applied in the field of the musculoskeletal system, e.g., in the evaluation of patient data, clinical decision support, and diagnostic imaging. In therapy, an artificial cognitive application was used to judge rehabilitation exercises based on the signals from the machine [113].

As a result of technological advancement, AI and robotics are transforming approaches and competencies in rehabilitation research and practice. For example, smart homes can assist residents with daily activities and alert caregivers when assistance is needed. In addition, smart mobile and wearable devices are available to collect data and provide users with information to assess health improvement and review progress toward personalized rehabilitation goals [114]. Additionally, inertial sensors in wearable technology can be recognized to check whether individuals are properly performing and adhering to exercise regimens [115]. Such exercise adherence was assessed in normal individuals following a rotator-cuff exercise regimen while wearing an Apple iWatch. Several supervised learning methods were applied to accurately categorize exercise accuracy across all algorithms [116]. A neural network achieved 99.4 percent detection accuracy, demonstrating the potential utility of wearable devices and ML in exercise tracking. However, assessing performance using wearable devices alone could possibly be insufficient to enhance adherence because of the variable snags to adherence connected with exercise efficacy [117,118]. Additionally, physically and socially supportive robots can be used to help individuals recover from injury or illness. These robots also support bridging gaps resulting from cognitive, motor, or sensory losses. These technologies play an important role in helping people increase their functional ability, independence, and healthy well-being [114]. Patients with musculoskeletal dysfunction were treated with simple mobilization using dextrous or soft robotic hands. However, the long-term efficacy of such treatment has yet to be demonstrated [119]. A recent study suggests that AI-enabled robotics could monitor the patient for perfection of movement and help the patient efficiently perform movements in the future [120]. Frackiewicz [121] stated that HCPs could match the gap between the need for rehabilitation services and the obtainability of trained therapists by adding an AI-driven tool, ChatGPT, to the rehabilitation sessions. Those can provide patients with an AI-driven method that complements conventional therapy. Using ChatGPT, the patients are provided with tailored and collaborative assistance; therefore, patients remain engaged and interested during their recovery process. ChatGPT can be programmed to deliver exercise recommendations and screen progress and offer feedback to individuals recovering from physical injuries. Further, it can also assist stroke or head injury patients in practicing their speech and language skills by involving them in conversation. This tool can be easily accessible using digital devices at home or outside. A recent study stated that a language model such as ChatGPT was trained to redraft text in a highly empathic way. It provides easy communication in a peer-to-peer mental health assistance system and improves non-expert conversational skills. It emphasizes the possibility of using human–AI partnerships to improve various community-based tasks relying on self- or peer-managed therapy, such as cognitive behavioral therapy [122]. 

Additionally, metaverse neurorehabilitation encompasses an AI-based gross motor function classification system (GMFCS), rehabilitation materials as a reward through rehabilitation, virtual character movement using weight shift, and deep learning-based movement assessment. It is planned to enhance interest and entertainment, deliver remedial exercises with AI, and limit the risk of COVID-19 transmission [123,124]. A recent study observed that metaverse physiotherapy (MPT) reduced perceived COVID-19 infection and enhanced cardiopulmonary and gross motor function compared with conventional physiotherapy in treating CP children [125]. In addition, AI has been used in gait analysis, where ML-driven video analysis demonstrated the ability of computers to automate the detection of gait abnormalities and related pathologies in patients with osteoarthritis and Parkinson’s disease [126]. Physiotherapy at home can be successfully delivered with counselling/advice, real-time observation, and remote tracking by digital therapists [120]. Lambercy et al. [127] envisioned an approach to delivering remote neurorehabilitation using digital interventions such as minimally supervised assisted therapy, which could help stroke victims continue care from the hospital and to their homes. However, AI-embedded technologies for remote neurorehabilitation should meet the technical needs, namely, utility, safety, and robustness, as the patients are trained with the devices at home, and their needs are met. These technologies should be scalable, and their application in neurorehabilitation requires a clinically motivated and transparent approach to patients and their families as well as HCPs. This condition could increase confidence in technology-enhanced rehabilitation in a home model. Additionally, all of these characteristics are critical to ensuring that patients with neurological disorders consent to technology-enhanced rehabilitation and actively engage in therapy [128]. Such technology-assisted rehabilitation at home notably impacted neurorehabilitation during and after the COVID-19 pandemic by providing widespread access to high-quality, sustained, and high-dose therapy that enhanced long-term functional outcomes and promoted independence and quality of life in stroke patients [127]. 

Concerning sports medicine, a recent review found that AI is a promising avenue for integration into wearable technologies. AI techniques processing data from sensors could monitor patterns in physiological measurements, as well as positional and kinematic data, to indicate how athletes can improve their performance. AI can improve how injury prediction models work, increase the diagnostic precision of risk stratification systems, provide a reliable technique for continuously monitoring patient health data, and improve the quality of patient experience. Although AI has beneficial applications in sports medicine, several challenges may hamper its adoption in wearable devices [129]. These challenges include missing data, socioeconomic bias, data security, outliers, signal noise, and the difficulty in obtaining high-quality data with wearable technology [129,130]. For example, sensors that monitor heart rate detect artifacts due to arm movements during physical tasks: Such a situation could be handled by highly complex sensors that could collect and transmit clean data [129]. Another critical challenge to their adoption is patient acceptance. Previous studies stated that half of consumers with a wearable device had stopped using it, and this condition had occurred within six months in one-third of them [131,132]. A previous study reported that half of patients felt that the use of AI in wearable technology was a significant opening, while 11% felt that it was harmful. Patients feared that the technology could exploit and misuse their data and affect the human aspect of healthcare. Therefore, patient education on how AI supports clinicians, and their abilities and limitations is needed to improve their acceptability and adoptability of AI [133]. 

### 2.6. Administrative Applications

AI can reduce administrative burdens by automatically populating structured data areas from therapeutic notes, retrieving key data from past medical records, and collecting documented patient encounters [134]. For example, the average nurse in the United States spends a quarter of his/her working hours on regulatory and administrative duties [135]. Physicians’ and nurses’ time could be saved using voice text writing [134]. Although rule-based systems integrated with electronic health record (EHR) systems are extensively used, they lack the accuracy of additional algorithmic systems based on ML [110]. Wang et al. [68] stated that Amazon is working on an innovative ML solution to extract valuable information from unstructured EHR data and scientific notes. Furthermore, Li et al. [32] introduced bidirectional encoder representations from a transformer for EHR (BEHRT), a deep neural sequence transduction model for EHRs. The BEHRT showed the ability of using various embeddings, such as age, position, visit, and event, to characterize the patient’s clinical history. It can concurrently forecast the probability of 301 conditions in an individual’s future visits. It significantly improves the average precision scores in various tasks compared with present deep EHR models. It can integrate various heterogeneous concepts, such as assessment, diagnosis, and drugs, due to its stretchy construction, thereby enhancing the accuracy of its predictions. A recent study proposed a hierarchical BEHRT (Hi-BEHRT) model, a hierarchical transformer-based model, for risk prediction. The study observed a significant performance of Hi-BEHRT compared with existing deep-learning models in risk prediction tasks for patients with a long clinical history of diabetes, heart failure, stroke, and chronic kidney disease [35].

Interestingly, Robotic Process Automation (RPA) can be used for various healthcare functions. These functions include clinical records, revenue cycle administration, claim handling, and medical record management [136]. Additionally, chatbots have been used by healthcare organizations (HCOs) for telehealth, mental health, and patient interfaces. These NLP-based tools can be helpful for simple transactions such as booking appointments or refilling prescriptions [137]. Regarding payment administration and claims, another AI technology, ML, can be applied to match data across different websites [134]. Insurance agencies are responsible for verifying the accuracy of many claims. Inappropriate claims slipping through the cracks demonstrate significant monetary potential waiting to be resolved using data reconciliation and claim scrutiny [109]. 

In addition, Corny et al. [138] concluded that a hybrid ML-based decision support system (ML rule-based expert system) was remarkably more accurate in detecting prescribing errors in a clinical setting. A recent review examined the development of AI tools for clinical pharmaceutical services and found that ML techniques and subsets, namely, NLP and deep learning, were widely used. It concluded that the growth of AI-based applications and tools for clinical pharmacy services is just beginning. Significant action needs to be taken in conjunction with data professionals to better assess whether these AI tools have value for clinical pharmacy services in real-world settings. Pharmacists need to recognize these gains in order to properly employ them while maintaining their social relationships with patients and healthcare teams [139]. 

## 3. Challenges Faced by AI Utilization in Healthcare

### 3.1. Ethical and Social Challenges

Several ethical and social disputes raised by AI overlap with those raised by high reliance on technology; automation; data usage and issues arising from the usefulness of ‘telehealth’ and assistive technologies, as the effectiveness of AI increases ethical concerns, including the issue of accountability when AI is used in decision making; the ability of AI to make erroneous judgments; AI yield authentication issues; the confirmation of the protection of sensitive data; intrinsic biases in the data used in AI system tests; maintaining public confidence in the growth and benefits of AI systems; influencing the sense of dignity and social isolation of the public in care settings; implications for HCPs’ roles and skill requirements; and the ability of AI to be used for malicious activity. Furthermore, safety and reliability problems may occur while using AI to deliver treatment, make decisions, and control healthcare equipment. AI could cause errors, and those might be challenging to detect or might induce adverse effects, which could lead to severe consequences [69]. For instance, the AI app predicting pneumonia-related complications mistakenly advised physicians to discharge patients with asthma because it failed to consider related information [140]. Concerning transparency and accountability, queries arise about the accountability of AI-made decisions and compensation for individuals affected by AI use. Issues related to AI output authentication and error or data bias recognition arise, especially with ML technologies, which could be mainly non-transparent due to the method whereby they constantly review their own limits and guidelines as they learn [69]. Additionally, explainability denotes a main obstacle facing AI regarding its practical implications in various domains. It is crucial and tackled by “Explainable artificial intelligence (XAI),” a branch of AI research, to overcome the poor understanding of AI-based applications and improve the acceptance of those applications concerning the decision-critical domain [141]. Furthermore, the internal mechanisms of AI are commonly non-transparent and complex for humans to comprehend. This condition can result in poor trust and comprehension concerning AI-made decisions. To overcome such issues, XAI is used to make the internal mechanisms of AI transparent and explainable to humans to create trust in and comprehension of AI-made decisions [142]. It is a pool of techniques that understand and trust the outcomes of ML algorithms [143]. In healthcare, doctors and patients can realize the reasons behind diagnosis using XAI, since it can explain diagnostic decisions made by AI [142]. A recent study found that XAI establishes trust in AI by giving visual feedback to the user regarding significant metrics that are used to obtain the model prediction [144]. Another study observed that XAI methods are required to improve radiologists’ trust in classification predictions of CT images with the acquisition of numerous visual insights into the automatic workflow [145]. 

Furthermore, AI might poorly function with data scarcity or difficulties in digital data collection. This state could impact the individuals understated in clinical trials or those with rare medical diseases [69]. While training AI applications using data, these applications can replicate and strengthen data biases even though those can decrease human error and bias [146]. Such data for training AI could poorly represent the broader populace and result in unfair decisions replicating more comprehensive biases in societies [70]. Those applications in healthcare also face data privacy and security challenges, because they utilize sensitive and private data bound by legal panels. Although AI can be applied for detecting cyber attacks and protecting healthcare desktops, there is the possibility for AI systems to be hacked to access sensitive data or spammed with biased or false data in such a way that might not be simply traceable [69]. Sunarti et al. [147] also listed the ethical issues faced by clinical AI applications: privacy, safety, security, the right to choose and try, cost, information and consent, access, and efficacy. Therefore, critical medical–ethical principles, such as beneficence, autonomy, equity, and non-maleficence, should be emphasized before integrating AI into healthcare systems [148].

### 3.2. Governance Challenges 

As the implementation of AI technologies in healthcare increases, there is a serious requirement for proper governance to overcome regulatory, ethical, and trust issues [149,150]. Active governance at the hospital level offers an opportunity to accurately address these issues in the implementation and use of AI [151]. Additionally, a recent study found that governing AI technologies at the healthcare system level is critical to patient safety and healthcare system accountability. Such governance also increases clinician confidence, improves acceptance, and makes significant health consequences possible. The governance structure should be comprehensive to address the challenges related to clinical, operational, and leadership domains while deploying AI-powered applications [152]. 

Additionally, AI has applications in areas that need regulation, including healthcare, research, and privacy. Nonetheless, AI is developing rapidly and commercially, which could challenge the known outlines [70]. National and international regulations are, therefore, required to introduce AI-controlled applications in healthcare as part of the principles of medical ethics. As such, the European Union (EU) developed general data protection regulation (GDPR), a data protection regulation in 2018, to control AI. GDPR shields the personal data dealt with by a data processor or controller recognized inside the EU. It is a base for essential reforms in the United States and Canada [153]. Recently, the revealed artificial intelligence act (AIA) was developed by the European Commission to address various risks related to the social adoption of AI. It is a set of regulations encouraging the acceptance of AI and intends to avoid or alleviate harms connected to specific usages of technology. According to the proposed act, high-risk AI systems must undergo pre-deployment conformity appraisals and post-market observational analysis to confirm that they meet all the necessities of AIA [154].

### 3.3. Technical Challenges 

Technically, AI models must be simple in their properties and functions in order for HCPs to efficiently operate them [155]. On the other hand, there are a few hurdles to adopting AI in healthcare, including the lack of capacity of developing and maintaining IT infrastructure to support the AI process, the increased costs associated with storing and backing up data for research purposes, and the high cost of augmenting data validity [156]. In addition, AI algorithms can suffer from a variety of shortcomings, including inapplicability outside of the training domain, bias, and brittleness (tendency to be easily fooled) [157]. Important factors to consider include dataset shifts, randomly matching confounders instead of true signals, the prevalence of unintended biases in clinical practice, providing interpretability for algorithms, developing reliable measures of confidence in the model, and challenging the generalization to different populations [158]. Therefore, healthcare providers should develop and implement an effective strategic plan for implementing AI in healthcare to address the issues related to cost, technological infrastructure, and the use of AI systems for HCPs. 

Additionally, HCPs often mistrust or poorly understand AI-grounded clinical decision support systems because of unidentified risks, a vital blockade to extensive adoption. In such circumstances, XAI solutions are emphasized to enhance end-user trust and overcome low AI adoption [144]. Additionally, Choudhury and Asan [159] found that factors such as risks and trustworthiness of AI, workload, and willingness to receive AI training influenced clinicians’ perceptions of the use of AI in healthcare. The lack of AI accountability has also been identified as an inhibiting element in using AI. It is recommended that AI training be included in medical and nursing curricula to enable AI to be safely used in the future. 

Similar to our brains, AI systems receive inputs and provide outputs. However, HCPs have no idea about what is being measured and how an AI system arrives at a result; all they know is the final output. This challenge in AI systems is called black-box problem [160]. Therefore, blaming healthcare practitioners for AI errors could be an obstacle to AI adoption, and it is recommended to develop policies and measures to protect doctors and AI in healthcare. This also emphasizes improving healthcare professionals’ risk perception and performance expectations of AI. It is crucial to ensure a user-friendly AI interface in which the information presented must have clinical relevance. There is a need to engage all stakeholders (providers, payers, and patients) and understand clinical needs before developing and promoting AI in healthcare.

## 4. Disadvantages of AI in Healthcare

Huge datasets are mandatory for machine learning and deep learning models to appropriately categorize or forecast various tasks. Nevertheless, the healthcare industry possesses a composite problem with data accessibility, since patient records are confidential and given the usual hesitancy among HCOs to exchange health data. In addition, data are not readily available once an algorithm is initially executed using them. Notably, ML-based systems can continually progress as additional data are provided to their training set; however, it is hard to attain this scenario due to internal corporate resistance [161]. Moreover, AI-based applications raise data security- and privacy-related issues. Hackers usually focus on health records during data breaches, since those records are significant and vulnerable. Hence, it is vital to uphold the confidentiality of health records [162].

Additionally, the overfitting issue occurs when the algorithm absorbs the connections between patient characteristics and results. This problem occurs due to numerous variables affecting the outcomes, which makes the algorithm make inaccurate predictions. Data leakage is another concern that describes AI’s ability to predict incidences beyond the diminished training dataset when the algorithm attains greater prediction accuracy [163,164,165]. Further, deep learning algorithms are less capable of giving substantial explanations for their predictions. An algorithm experiences difficulty in protecting itself legally when recommendations go wrong. It makes it difficult for experts to comprehend how the data are associated with their forecasts. The black-box issue in AI systems may lead the public to lose faith in healthcare systems [166].

The healthcare workforce may fear AI in healthcare, which might reduce their occupations and replace them. At the same time, they are in need to be re-engineered. Another issue with AI is the price tag covering the time and resources invested in training HCPs to successfully use AI [161]. In healthcare, insufficient experimental data authenticating the efficacy of AI-based drugs in planned clinical trials is a critical hindrance to the positive employment of AI. AI research has been mainly performed in non-clinical environments. Hence, the generalization of findings might be difficult. Similarly, the institutions are uncertain and reluctant to execute AI-based solutions because of the lack of empirical data and the rutted quality of research [167]. Other disadvantages of AI, in general, include the high costs of creating AI-based applications, making humans lazy, creating unemployment due to replacing repetitive tasks with AI, and a lack of emotions and creativity in machines [168]. 

## 5. Conclusions

AI technologies are being used for a range of healthcare applications. These technologies have been developed to support medical imaging and diagnostic services, fight the pandemic, provide virtual patient care, increase patient engagement and adherence to treatment plans, reduce the administrative burden on healthcare professionals, drive drug and vaccine innovation, monitor the compliance of patients with exercises, and carry out gait analyses used in technology-assisted rehabilitation. However, AI also faces various technical, ethical, and governance challenges as it moves forward in healthcare. It raises data security- and privacy-related issues because it utilizes sensitive and confidential data bound by legal panels. The use of AI in addressing challenges could be limited by the quality of existing health data and AI’s failure to reflect certain human characteristics, such as compassion. AI is more beneficial while functioning efficiently; however, it cannot replace the human connections that form teams. Human functions such as teamwork and team management are not possible-to-achieve goals, since machines cannot form a bond with humans. A key challenge to be solved for the future governance of AI technologies will be to confirm that AI can be developed and implemented in a way that aligns with people’s interests and takes into account technical, ethical, and social aspects. This study adds to the existing literature compiling the application of AI in medical imaging and diagnostics, virtual patient care, medical research and drug discovery, patient engagement and adherence, rehabilitation, and other administrative applications. Additionally, this is the latest update in the literature to address the ethical, social, governance, and technical challenges that HCPs face in adopting AI in healthcare.

### Future Directions 

Since this study is based on a general literature review, future research could focus on conducting a more comprehensive systematic literature review that can provide a deeper insight into this research topic. In addition, future research should focus on a cross-sectional survey of HCPs to collect primary data on all the key issues addressed in this study.

## Figures and Tables

**Table 1:** Definition of terms related to AI.

Term	Definition
Artificial intelligence (AI)	AI denotes the science and engineering of creating intelligent machines using algorithms or rules, which the machine shadows to mimic human cognitive functions, namely, learning and problem solving [13]. AI usually refers to computer technologies that emulate mechanisms supported by human intelligence, namely, adaptation, deep learning, reasoning, engagement, and sensory understanding [14,15]. It aims to mimic human cognitive functions. It brings a paradigm shift in healthcare, driven by the increasing availability of health data and the rapid growth of analytical techniques [16].
Machine learning (ML)	ML is a subtype of AI technology that aims to improve the speed and accuracy of physicians’ work. It also denotes several statistical techniques that allow computers to learn from experience without being explicitly programmed. This learning usually takes the form of variations in how an algorithm works [17]. It is also a tool applied in healthcare to assist healthcare professionals in caring for patients and managing clinical data. It is an application of AI that involves programming computers to mimic how humans think and learn [18].
Distributed Ledger Technology (DLT)	DLT is an innovative and rapidly growing method for recording and sharing data across different data stores (ledgers) [19]. It is secure, immutable, and readily available. It can allow patients to take control of their own data, eventually generating trust in an industry that matters to all of us [20]. DLT integrated with AI describes a novel and advanced method to achieve the intelligent, resilient, and safe handling of electronic health record data [21].
Natural language processing (NLP)	Natural language processing (NLP) denotes the field of study that emphasizes the interactions between human language and computers [22]. NLP techniques can capture unstructured healthcare information, analyze its grammatical structure, determine the meaning of information, and translate information; therefore, it can be easily understood by electronic healthcare systems. These techniques also reduce costs and improve the quality of healthcare [23].
Metaverse	The metaverse represents a 3D space based on virtual and augmented reality, where individuals can utilize their own avatars to play, work, and synchronously interconnect with each other [24]. It delivers an entrancing, communicative, and pleasurable healthcare service experience tailored to achieve patients’ desires. It includes modern technologies such as AI, telepresence, blockchain, virtual reality (VR), augmented reality (AR), and digital twinning. These technologies highly influence healthcare [25]. The metaverse application is exclusively associated with healthcare, establishing a “niche theme” for academics, such as education, research, training, and disease prevention and management. It has become a vibrant technology for strengthening medical students’ competence. Furthermore, patients’ health illnesses can be directly monitored at their homes, and real life can also be connected with the virtual one using digital twins, a diverse technology [26,27].
Chat Generative Pretrained Transformer (ChatGPT)	ChatGPT is an AI-based conversational agent that utilizes natural language processing (NLP) and machine learning algorithms to simulate human-like conversations [28]. Its critical applications in healthcare, including practice, education, and research, could be auspicious if the accompanying valid concerns are proactively inspected and tackled. It functions as a chatbot, a program that can comprehend and create responses using a text-grounded interface [29]. Xu et al. [30] described the application of chatbots in healthcare, comprising patient support; monitoring and administration; and tumor diagnostics, screening, and management.
Transformer	Transformer is a critical deep learning model and is broadly used in various areas, namely, computer vision (CV), natural language processing (NLP), and speech processing [31]. The applications of transformers are observed in electronic health records, medical imaging, and COVID-19 detection [32,33,34,35,36].

## Data Availability

Not applicable.
